# Fucoidan-based coatings extend the shelf-life of nectarines

**DOI:** 10.1016/j.fochx.2024.101479

**Published:** 2024-05-17

**Authors:** Yusi Lan, Yu Liu, Xiang Li, Shengjun Wu

**Affiliations:** a Jiangsu Key Laboratory of Marine Bioresources and Environment/Jiangsu Key Laboratory of Marine Biotechnology, Jiangsu Ocean University, 59 Cangwu Road, Haizhou 222005, China; b Co-Innovation Center of Jiangsu Marine Bio-industry Technology, 59 Cangwu Road, Haizhou 222005, China

**Keywords:** Nectarine, Fucoidan, preservation

## Abstract

This research investigated the efficacy of fucoidan-based coatings in preserving nectarine fruits at room temperature. The present study compared the preservation effects of different fucoidan concentrations (1%, 3%, 5%) with distilled water serving as a control (0%). The findings revealed that the addition of fucoidan dose-dependently improved the room temperature preservation quality of the nectarines. Notably, a 5% fucoidan concentration markedly delays the onset of the respiratory peak in nectarines. On day 14 of storage, the plants were subsequently cultured on a 5% fucoidan coating (F5), which exhibited a weight loss rate of 5.87%, a spoilage rate of 18.33%, a hardness of 3.87 kg/cm², a soluble solid content of 11.47%, a titratable acid content of 0.29% and an ascorbic acid content of 2.58%. The overall acceptability score was 7.83. These results demonstrated that coating with fucoidan is an effective method for the preservation of nectarines.

## Highlights

• The coatings suppressed respiration rate and weight loss.
• The coatings suppressed decay incidence and maintained firmness.
• The coatings suppressed the decreases in soluble solid and titratable acid content.
• The coatings suppressed the decrease in ascorbic acid content.
• The coatings suppressed the decrease in likeness.

## 1 Introduction

Nectarine plants are fruits of the Rosaceae plant family and are characterized by smooth and hairless skin, vibrant color, a sweet and sour taste, a rich fruity aroma, and high levels of vitamin C, flavonoids, phenols, carotenoids, and other nutrients (Drogoudi et al., 2016; Gil Maria et al., 2002; Kumari, Karanjeev, Sanjay, & Feza, 2020; Wang, Hao, Zhou, Pan, & Tu, 2022). China is a major producer of nectarines, with peach and nectarine planting areas estimated to be 865,100 hectares in 2022, yielding 16,817,100 tons. Due to their appealing taste and nutritional value, nectarines have gained popularity among consumers and are considered important economically along with apples and pears (Obi, Barriuso, & Gogorcena, 2018; Weisskopf & Fuller, 2014). However, as a climacteric fruit, nectarines are vulnerable to postharvest decay and loss of nutritional and economic value during storage (Cao, Zhou, Ge, Luo, & Su, 2022; Liu et al., 2024). Therefore, finding effective methods to prolong the postharvest storage time and reduce losses is crucial. Various physical and chemical methods, such as controlled atmosphere treatment, hot water immersion, low-temperature storage, and the use of 1-methylcyclopropene, have been explored to improve storage quality and extend shelf life (Akbudak & Eris, 2004; Jemric et al., 2010; Özkaya, Yildirim, Dündar, & Tükel, 2016; Sisquella, Casals, Viñas, Teixidó, & Usall, 2013). Nevertheless, these methods often have drawbacks, including complex operation, high cost, sensory quality alteration, and potential safety hazards. Consequently, there is a need in the market and among consumers for a green, safe, cost-effective, and user-friendly method for nectarine preservation and storage. Edible coating treatment, specifically a film preservation technique, has emerged as a viable solution. The natural essential oils chitosan, sodium alginate, pectin, sodium caseinate, and other substances derived from animals and plants have been employed as edible coating materials for nectarine preservation and storage (Cao et al., 2022; Chiabrando & Giacalone, 2016; Ramirez, Timón, Petrón, & Andrés, 2015; Zhang et al., 2019). Fucoidan, also referred to as fucoidan sulfate, is a naturally occurring polysaccharide abundant in sulfate groups. It is commonly found in various types of brown algae, such as focus, kelp, and sargassum. Additionally, fucoidan can also be found in marine mollusks, such as sea cucumbers (Ana et al., 2021; Fitton, Stringer, & Karpiniec, 2015; Ramirez et al., 2015; Thao My et al., 2020). Fucoidan is structurally heterogeneous and does not possess a specific unified structure. It has a wide range of physiological activities, including antioxidant, antiviral, antibacterial, immunomodulatory, anticoagulant, anti-inflammatory, antithrombotic, and free radical scavenging properties (Biswajita et al., 2022; Cátia et al., 2022; Dore et al., 2013; Li et al., 2022; Paolina, Elisaveta, Alexandra, Marianna, & Vesela, 2023; Wang et al., 2022). Due to its natural safety and diverse physiological activities, fucoidan has attracted increased interest in research. Studies have explored the use of fucoidan as an edible polysaccharide coating for the low-temperature and room-temperature preservation and storage of fruits such as strawberry, cucumber, and mango plants (Lin et al., 2022; Luo, Li, Liu, Yang, & Duan, 2020; Xu & Wu, 2021). However, there is limited information available regarding the application of fucoidan in the preservation and storage of nectarines. Fucoidan is a natural water-soluble polysaccharide, and nectarines are perishable fruits. Therefore, exploring the preservation effect of fucoidan not only meets consumer expectations for food safety, but also is greener and more convenient compared to other preservation methods.

The objective of this study was to examine the effect of fucoidan on the preservation and storage of nectarines at room temperature. This goal will be achieved by coating nectarine fruits with different concentrations of fucoidan solutions and regularly measuring the corresponding quality and nutritional indicators of the nectarines throughout the storage period. The aim is to discover novel approaches for preserving and storing nectarines.

## 2 Materials and methods

### 2.1 Materials

Nectarines were procured from the agricultural product market in Lianyungang city, Jiangsu Province, China. Experimental samples were selected based on criteria such as a maturity level of 70% to 80%, intact fruit shape, uniform color, similar size, absence of pests and diseases, and no mechanical damage. Food-grade fucoidan with a purity of 98%, a chemical formula of (C_6_H_10_O_7_S)_n_ and a molecular weight of 8.2 kDa was obtained from Qingdao Gongying Marine Biotechnology Co., Ltd. (Shandong Province, China). All the other chemicals used were reagent grade (China National Pharmaceutical Group Corporation, Beijing, China).

### 2.2 Coating treatment and storage conditions for the nectarine fruits

Preparation of the fucoidan coating solution: The distilled water was utilized to prepare fucoidan solutions at various concentrations (1%, 3%, 5%), while distilled water was served as a control coating solution at a concentration of 0%. After naturally washing and drying, the nectarines were randomly divided into four groups, each containing 120 nectarines. Subsequently, the divided nectarines were immersed in fucoidan coating solutions at concentrations of 0%, 1%, 3%, and 5% for 5 min. Once the immersion process was completed, the nectarines were removed for natural drying. The samples were then stored at room temperature, specifically at 20 °C with 80% humidity, and kept away from light for 14 days. The treatment groups were denoted F0, F1, F3, and F5, with F0 designated the control group. Throughout the nectarine storage period, samples were collected every two days to evaluate their quality and nutritional indicators.

### 2.3 Determination of the respiration rate

In accordance with the approach developed by Xu and Wu (2021), nectarine fruits weighing approximately 1 kg were randomly selected and placed into a 1 L glass container. Subsequently, the container was sealed and heated at 25 °C for 1 h. A gas chromatograph and thermal mass spectrometer from the Trace 2000 GC series were used to analyze the CO_2_ content within the headspace sample at different time intervals. The respiration rate is expressed as CO_2_ production, in mg/(kg·h).

### 2.4 Determination of the weight loss rate

On day 0 of the experiment, the nectarine fruits within control group (F0) and experimental group (F1, F3, F5) were weighed, and the weight was recorded as w_0_ (g). Subsequently, the nectarine fruits in each group were weighed again every two days, and the new weights were recorded as w_1_. The weight loss rate was calculated as follows: weight loss (%) = (w_0_-w_1_)/w_0_×100%.

### 2.5 Determination of decay incidence

The initial number of nectarine fruits (g_0_) in each group was recorded. The number of rotten nectarine fruits (g_1_) in each group was recorded every two days. The decay incidence rate was calculated as follows: spoilage rate (%) = g_1_/g_0_ × 100%.

### 2.6 Determination of firmness

The nectarine fruit firmness was measured using a handheld fruit hardness tester (GY-3 type, Wenzhou Yiding Instrument Manufacturing Co., Ltd, China). After removing the peel on both sides along the longitudinal centerline of the nectarine, a GY-3 fruit hardness tester with a 3.5 Nm probe was used to measure the hardness at three different points within the center of the pulp. The unit of measurement is kg/cm^2^.

### 2.7 Determination of total soluble solids content

The nectarine fruits were homogenized (FSH-2A, Changzhou Yuexin Instrument Manufacturing Co., Ltd, China), filtered, and subjected to centrifugation (TG 16G, Jiangsu Lingli Technology Co., Ltd, China). The resulting supernatant was utilized to determine the total soluble solid content using a refractometer (WAY-Z, Shanghai Nengong Industrial Co., Ltd., Shanghai, China).

### 2.8 Determination of titratable acid content

In accordance with the methods of Naeem, Abbas, Ali, and Hasnain (2018), the nectarine fruits were homogenized, filtered, and centrifuged. The resulting nectarine fruit supernatant was titrated with a NaOH solution at a concentration of 0.1 mol/L. The titratable acid content was calculated using the following equation: TA = (v × 0.067 × 0.1 × 100)/m, where v represents the consumption volume of the NaOH standard solution (ml) and m is the mass of the homogenized supernatant (g). The constant 0.067 is the malic acid conversion coefficient.

### 2.9 Determination of ascorbic acid (AA) content

In accordance with the methodology of Liu et al. (2014), nectarine fruits were homogenized (FSH-2A, Changzhou Yuexin Instrument Manufacturing Co., Ltd, China), filtered, and centrifuged (TG 16G, Jiangsu Lingli Technology Co., Ltd, China) to prepare a supernatant sample liquid. The sample liquid was then mixed with 4 mL of oxalic acid-EDTA solution, 1.5 mL of 3% metaphosphoric acid-acetic acid solution, 2 mL of 5% sulfuric acid solution, and 2 mL of 5% ammonium molybdate solution. The mixture was diluted to 25 mL with ultrapure water, and the mixture was incubated in a 30 °C water bath for 20 min. After cooling to room temperature, the absorbance was measured at 700 nm, after which the AA content was calculated. Calibration curve (y=9.781x − 0.0495; R^2^=0.9991), record using standard vitamin C solution within the range of 2-22 μg/mL. The content of ascorbic acid is expressed as milligrams (mg/100g) of ascorbic acid equivalent per 100 grams of fresh avocado weight.

### 2.10 Sensory evaluation

According to the methodology of Xu and Wu. (2021), during the storage period of nectarines at room temperature, samples of nectarine fruits were collected, peeled, and sliced every 2 days. The samples were evaluated by 30 professionally trained students from the College of Marine Food and Biotechnology of Jiangsu Ocean University, Lianyungang City, Jiangsu Province, China. The overall acceptability of the nectarine fruits from the experimental and control groups was assessed using a descriptive hedonic scale ranging from 1 to 9. A score of 1 represented the lowest sensory quality, while a score of 9 represented the highest sensory quality. This experiment was approved by the ethics committee of Jiangsu Ocean University, China. All procedures were conducted in compliance with relevant laws and institutional guidelines. A recording statement of consent is available to confirm that the participants gave their consent to take part and use their information.

### 2.11 Data analysis

All the experiments were performed in triplicate. The data are expressed as the mean ± standard deviation. Data collection and analysis were conducted using Origin 7.0 and SPSS 27.0.1 statistical analysis software. A paired samples t test was used to analyze significant differences.

## 3 Results and discussion

### 3.1 Respiration rate

**Fig. 1:**
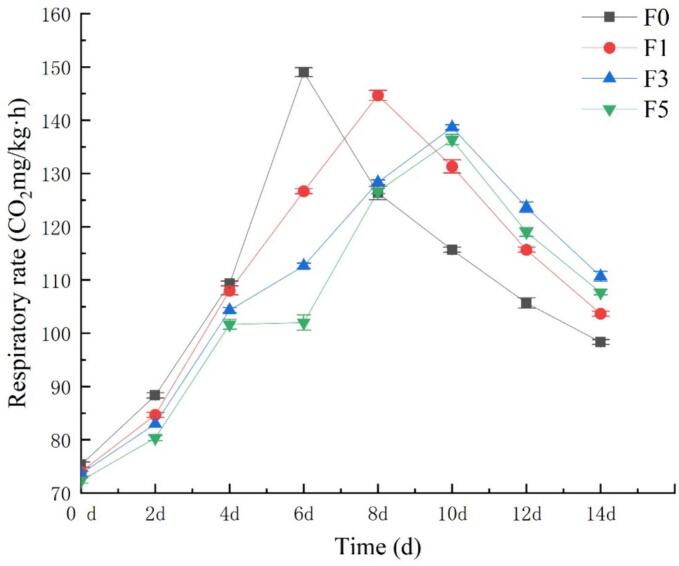
Effect of fucoidan coating on the respiration rate of nectarines stored at room temperature. Values are three repeated values. The vertical bar represents the standard deviation.

After harvesting, fruits continue to consume their nutrients for respiratory metabolism. Therefore, the respiration rate serves as an important indicator for evaluating the freshness and nutrient consumption rate of stored and preserved fruits (Pieczywek, Nosalewicz, & Zdunek, 2024). Effectively inhibiting the respiratory metabolism of postharvest fruits can significantly prolong their preservation time. Nectarines, which are climacteric fruits, exhibit noticeable respiratory peaks during storage. As shown in Fig. 1, the postharvest respiration rate of the nectarine fruits in all treatment groups initially increased and then decreased. The control group (F0) reached its peak respiration rate on the 6th day of storage, followed by a rapid decrease. However, the respiratory activity of the nectarine fruits treated with the fucoidan coating was inhibited to varying degrees, resulting in a delayed peak appearance and a slower rate of decrease compared to those in the control group. The respiratory potential of the nectarine fruits treated with the 1% fucoidan coating peaked on the 8th day, while those treated with the 3% and 5% fucoidan coatings reached their respiratory peak on the 10th day. The results indicate that the F5 treatment had the greatest inhibitory effect on the postharvest respiratory intensity of the nectarine fruits, followed by the F3 treatment. Compared with the other groups, experimental group F1 had a lesser inhibitory effect on fruit quality, suggesting that a 5.0% fucoidan coating yields better preservation results for processed nectarine fruits. This can be attributed to the formation of a transparent film on the surface of the nectarine fruit after fucoidan coating treatment, which regulates gas exchange inside the fruit, thereby inhibiting respiration. The films formed by coating with different concentrations of fucoidan also resulted in varying degrees of changes in the CO_2_ and O_2_ contents within the fruit (Xu & Wu, 2021).

### 3.2 Weight loss rate

**Fig. 2:**
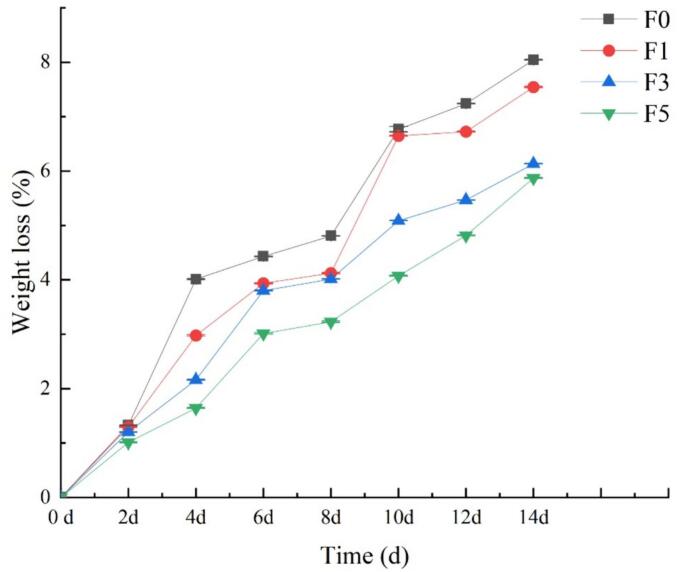
Effect of fucoidan coating on the weight loss rate of nectarine fruits stored at room temperature. Values are three repeated values. The vertical bar represents the standard deviation.

Nectarine fruits have a high water content during the early postharvest stage. Due to transpiration and respiratory metabolism, plants gradually lose water and wilts, thereby impacting their appearance, quality, and taste (Jiang, Feng, & Li, 2011). Consequently, reducing water loss during the preservation and storage of nectarines is crucial for ensuring their storage quality. Fig. 2 shows that the % weight loss of the nectarine fruits was positively correlated with the storage time. In other words, the longer the storage time was, the greater the weight loss rate of the fruit. However, the weight loss rate of the nectarine fruits treated with the fucoidan coating was significantly lower than that of the control group treated with distilled water during storage. Furthermore, the efficacy of the fucoidan coating in inhibiting water loss in nectarine fruits increased with increasing concentration. Notably, the rate of fruit weight loss was significantly lower in the F5 experimental group than in the control group and the other two experimental groups (*P* < 0.05). These results indicate that fucoidan coating helps maintain an optimal moisture content in nectarine fruits, delays fruit decay, and notably preserves fruit. This can be attributed to the formation of a dense preservation coating on the nectarine fruit surface, which possesses excellent water retention properties, effectively locking in fruit moisture, enhances the fruit's antioxidant capacity, and inhibits postharvest fruit physiological metabolism and nutrient loss caused by transpiration (Lin et al., 2022). Consequently, this approach helps create a stable internal environment for nectarine fruit.

### 3.3 Decay incidence

**Fig. 3:**
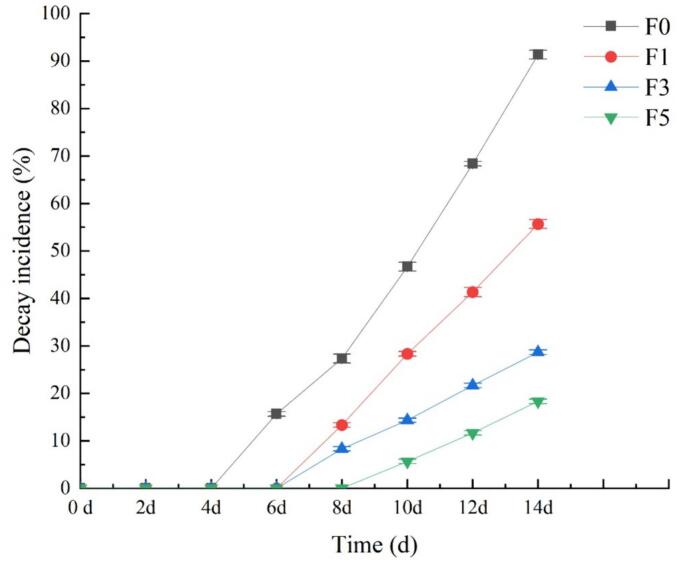
Effect of fucoidan coating on fruit decay incidence of nectarines stored at room temperature. Values are three repeated values. The vertical bar represents the standard deviation.

The spoilage rate serves as a key indicator for evaluating the effectiveness of postharvest storage and preservation of fruits, especially nectarine fruits (Jiang et al., 2011; Mou et al., 2023). Fig. 3 clearly shows that the size of the nectarine fruits gradually deteriorates as the storage period increases. On the 6th day of storage, the control group F0 plants treated with distilled water showed signs of spoilage. By the 8th day of storage, the plants in the experimental groups (F1, F3, and F5) also exhibited spoilage, with F5 consistently experiencing a lower rate of spoilage than the other three groups. By the 14th day of storage, the spoilage rate in the F0 treatment reached 91.33%, that in the F1 treatment reached 55.67%, that in the F3 treatment reached 28.67%, and that in the F5 treatment was 18.33%. These findings indicate that using a fucoidan coating during storage contributes to inhibiting nectarine spoilage, with the 5% concentration of fucoidan coating yielding the best preservation effect. Respiratory metabolism, microorganisms, and other factors strongly impact the susceptibility of nectarine fruits to rot and deterioration during storage, thereby diminishing their edible quality and shortening their shelf life. Hence, reducing the rate of decay is crucial for preserving nectarines. The efficacy of the fucoidan coating in effectively inhibiting the decay of nectarine fruits can be attributed to two factors. First, the dense coating created by the fucoidan coating on the fruit surface effectively suppresses respiration and minimizes the risk of microbial infection. Second, fucoidan itself possesses antibacterial properties, thereby helping fruits resist microbial infection (Ayrapetyan et al., 2021).

### 3.4 Fruit firmness

**Fig. 4:**
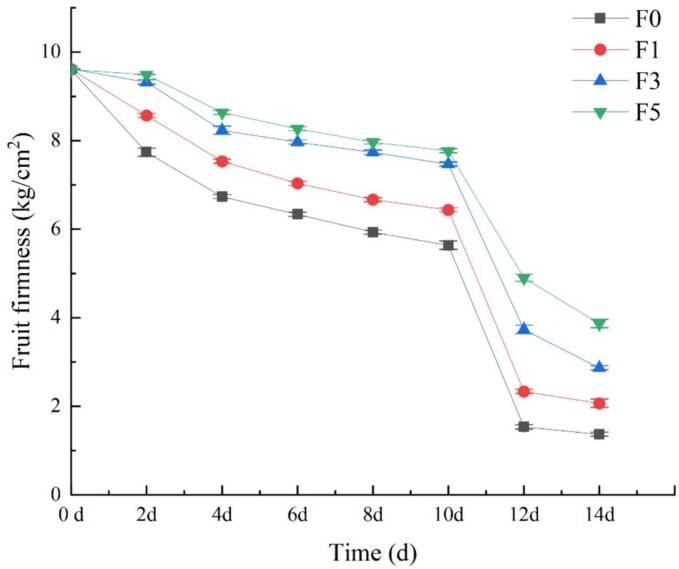
Effect of fucoidan coating on fruit firmness of nectarines stored at room temperature. Values are three repeated values. The vertical bar represents the standard deviation.

Fruit firmness is a critical parameter for assessing fruit freshness (Lobos, Retamales, Luengo Escobar, & Hanson, 2020; Rey-Serra, Mnejja, & Monfort, 2021). As depicted in Fig. 4, the hardness of the nectarine fruits decreased steadily with prolonged storage time. However, the firmness of the nectarine fruits in the experimental group treated with the fucoidan coating was lower than that of the fruits in the control group. After 10 days of storage, the firmness of the nectarine fruits in all the groups sharply decreased, possibly due to the late storage period, the onset of the respiratory peak, and the worsening of fruit softening. The results demonstrated that utilizing a fucoidan coating contributed to improved preservation of nectarine firmness, with the 5% fucoidan coating proving most effective at delaying the decrease in firmness. This favorable result can be attributed to the fact that the fucoidan coating forms a protective film on the fruit surface, thereby reducing the respiration rate, transpiration, water loss, and oxidation.

### 3.5 Total soluble solids

**Fig. 5:**
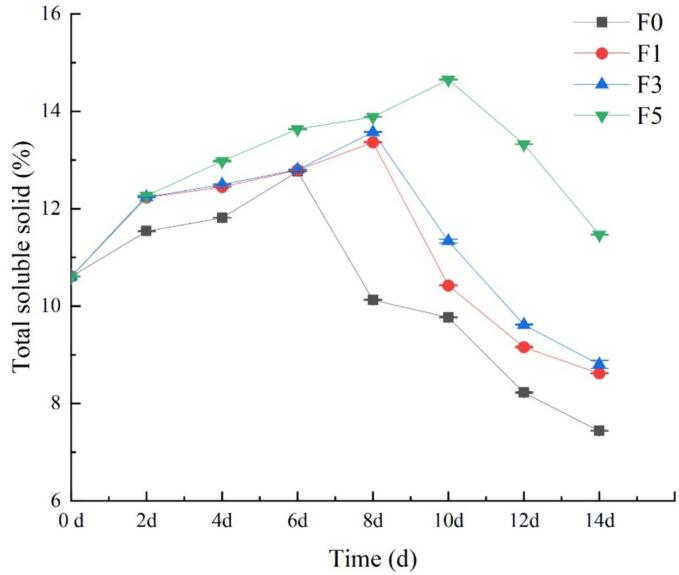
Effect of fucoidan coating on the total soluble solid of nectarine fruits stored at room temperature. Values are three repeated values. The vertical bar represents the standard deviation.

The nutritional profile, taste, and flavor of nectarine fruits are directly influenced by the soluble solids content. During early storage, the fruit's starch is converted into water-soluble sugars, thereby increasing the content of soluble solids (Naeem et al., 2018). As the storage time advances past the respiratory peak, some sugars become substrates for respiration, resulting in a decrease in soluble solid content. Fig. 5 shows that the soluble solid content in all treatment groups initially increased and then decreased with increasing storage time. Notably, the soluble solids content was consistently greater in the nectarine fruits treated with the fucoidan coating than in those in the control group. On the 14th day of storage, the soluble solids content of all the fruits in the experimental groups surpassed that of the control group (*P* < 0.05). Specifically, the 5% fucoidan coating effectively retained the soluble solid content of the fruit and preserved the quality of the nectarine fruit during storage. This difference may be attributed to the ability of the fucoidan coating to reduce the respiration rate of nectarine fruits.

### 3.6 Titratable acid content

**Fig. 6:**
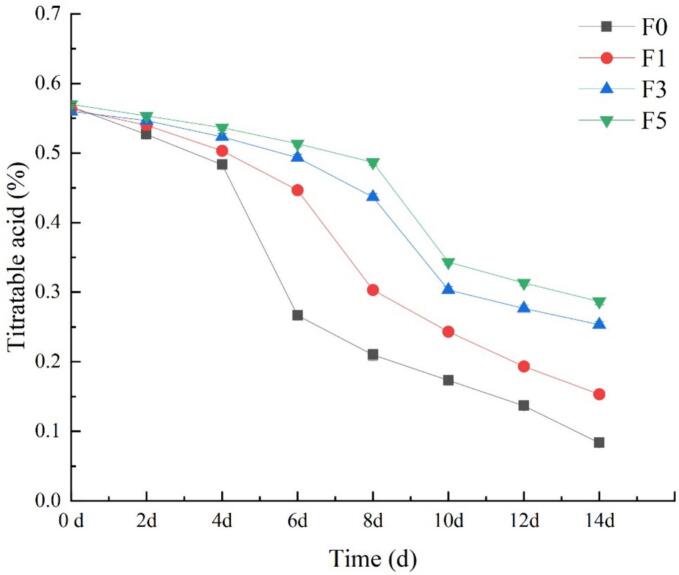
Effect of fucoidan coating on titratable acid content of nectarine fruits stored at room temperature. Values are three repeated values. The vertical bar represents the standard deviation.

Acidity represents one of the primary flavors of nectarines. The titratable acid content significantly influences the quality, taste, and flavor of plants and serves as an essential indicator of maturity. During the storage and ripening period of fruits, physiological processes such as respiration and metabolism will continue, converting organic acids into sugars to provide energy (Antoine et al., 2016; Aubert, Bony, Chalot, Landry, & Lurol, 2014). Therefore, the content of titratable acids will decrease during this period. Fig. 6 highlights that the titratable acid content of the nectarine fruits in each treatment group gradually decreased during storage, with the titratable acid content in the control group (F0) exhibiting a faster decline than that in the other three experimental groups. Concurrently, at the conclusion of the experiment, the titratable acid contents of the F1, F3, and F5 experimental groups were considerably greater than that of the control group F0, with increases of 87.5%, 212.5%, and 262.5%, respectively. These findings demonstrated that fucoidan coating treatment inhibited the reduction in titratable acid content in nectarine fruits, particularly when a 5% concentration of fucoidan was used to effectively maintain the titratable acid concentration. This effect may arise from the fucoidan coating reducing nectarine respiration and other metabolic rates, thereby slowing the decomposition of titratable acids into CO_2_ and water and preserving the fruit's titratable acid content and taste.

### 3.7 Ascorbic acid content

**Fig. 7:**
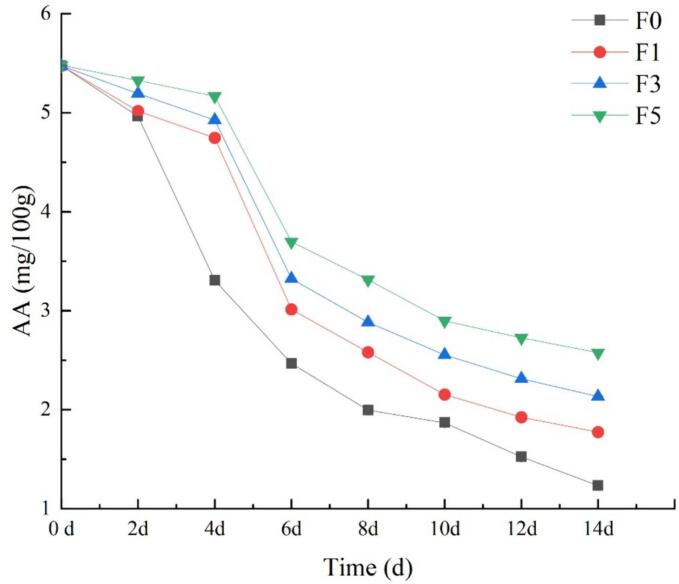
Effect of fucoidan coating on ascorbic acid content of nectarine fruits stored at room temperature. Values are three repeated values. The vertical bar represents the standard deviation.

The ascorbic acid content is a significant indicator that reflects the nutritional value of fruits (Deng et al., 2022). During the storage of nectarines, the ascorbic acid present in the fruit is highly unstable and susceptible to oxidation and decomposition, leading to a loss of nutritional value. Fig. 7 clearly demonstrates a downward trend in both the ascorbic acid content and titratable acid content of the nectarine fruits in each group. Notably, the ascorbic acid content of the experimental group treated with the fucoidan coating decreased more slowly than that of the control group. Moreover, the ascorbic acid content in the fruits of the experimental group remained higher than that in the fruits of the control group by the end of storage. In particular, the application of a 5% fucoidan coating treatment was more effective at maintaining the ascorbic acid content in nectarines. These findings suggest that the fucoidan coating can protect against ascorbic acid in fruit by preventing its decomposition and loss in a dose-dependent manner. This protective effect may be attributed to the film created by the fucoidan coating on the fruit surface, which establishes a high CO_2_ and low O_2_ environment within the fruit, effectively inhibiting the oxidative decomposition of ascorbic acid. Additionally, fucoidan itself exhibits antioxidant activity, further contributing to its protective effect.

### 3.8 Overall likeness

**Fig. 8:**
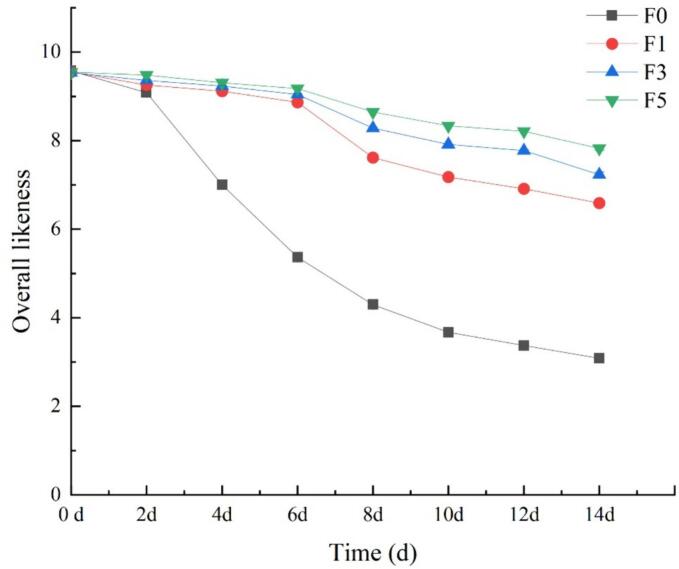
The effect of fucoidan coating on the overall acceptability score of nectarine fruits stored at room temperature. Values are three repeated values. The vertical bar represents the standard deviation.

Fig. 8 reveals that in the early stages of storage, there were no notable differences in overall likeness between the groups. However, after the sixth day of storage, the overall likeness of the control group F0 began to decline to an unacceptable level. Moreover, at the end of the storage period, the overall likeness of the F0 group decreased significantly by 67.82% compared to that in the initial stage of storage, while the fucoidan-treated groups still exhibited a significantly greater overall likeness than did the control group (*P* < 0.05) and remained within the acceptable range. This difference may be attributed to the barrier-forming ability of the fucoidan coating and its inherent antibacterial and antioxidant activities.

## 4 Conclusion

During the storage process, several factors contribute to the decline in the quality of stored nectarine fruits, including respiratory metabolism, transpiration, and microbial infection. However, applying a fucoidan coating treatment can create a dense preservation film on the surface of the fruits, resulting in various beneficial effects. These effects include a significant reduction in transpiration, respiratory metabolism, weight loss, and spoilage during storage at room temperature (20 °C, with a humidity of 80%). Additionally, this treatment also helps mitigate the loss of fruit nutrients, thereby enhancing the overall storage quality, flavor, and taste of the nectarine fruits. As a result, the fucoidan coating treatment proves to be an effective method for preserving the freshness of nectarine fruits and extending their shelf life. Moreover, this approach is environmentally friendly, safe, and highly efficient, representing a viable solution for keeping nectarines fresh at room temperature.

## CRediT authorship contribution statement

**Yusi Lan:** Writing – original draft, Methodology, Investigation, Formal analysis, Data curation. **Yu Liu:** Writing – original draft, Visualization, Validation, Software, Investigation. **Xiang Li:** Writing – review & editing, Supervision, Project administration, Investigation. **Shengjun Wu:** Writing – review & editing, Resources, Funding acquisition, Conceptualization.

## Declaration of competing interest

The authors declare that they have no known competing financial interests or personal relationships that could have appeared to influence the work reported in this paper.

## Data Availability

The authors do not have permission to share data.
